# Willingness to Become a Living Kidney Donor to a Stranger Among Polish Health Care Professionals Employed in a Dialysis Center: A National Cross-Sectional Study

**DOI:** 10.3390/jcm14155282

**Published:** 2025-07-25

**Authors:** Paulina Kurleto, Irena Milaniak, Lucyna Tomaszek, Wioletta Mędrzycka-Dabrowska

**Affiliations:** 1 Faculty of Health Sciences, Andrzej Frycz Modrzewski Krakow University, 1 Herlinga-Grudzińskiego Street, 30-705 Kraków, Poland; pkurleto@uafm.edu.pl (P.K.); imilaniak@uafm.edu.pl (I.M.); ltomaszek@uafm.edu.pl (L.T.); 2 Institute of Tuberculosis and Lung Diseases, Rabka-Zdrój Branch, Profesora Rudnika 3B Street, 34-700 Rabka-Zdrój, Poland; 3 Department of Anaesthesiology Nursing & Intensive Care, Faculty of Health Sciences, Medical University of Gdansk, Debinki 7, 80-211 Gdańsk, Poland

**Keywords:** living kidney, dialysis center, healthcare, medical personnel

## Abstract

**Background**: Kidney transplantation from a living donor is considered the most beneficial form of treatment for end-stage renal failure, which, in addition to providing patients with better treatment results, significantly improves their quality of life. Understanding factors that influence the willingness to donate kidneys to strangers is critical in promoting and expanding the living donor pool. When considering the decision to become an altruistic kidney donor, individuals must evaluate multiple factors, including the identity of the recipient and their own perceived level of safety. This study aimed to assess the willingness of dialysis center employees to act as living kidney donors for a stranger. **Methods**: We conducted a cross-sectional study from February 2023 to June 2024 among dialysis specialists across Poland. The study involved 1093 people (doctors and nurses). The study used our survey questionnaire and standardized tools. **Results**: Nurses (vs. physicians) and those who advocated the regulation of unspecified living kidney donation in Poland, did not believe in the risk of organ trafficking, and would donate a kidney to a husband/wife or friend and accept kidney transplantation from a husband/wife were more likely to donate a kidney to a stranger. Furthermore, respondents who accepted a loved one’s decision to donate a kidney to a stranger were significantly more willing to donate a kidney to such a person themselves. Perceived self-efficacy was positively associated with the willingness to donate a kidney to a stranger. **Conclusions**: Less than half of healthcare professionals supported unspecific living organ donation in Poland, and nurses were more willing to donate than physicians. The factors supporting the decision generally included knowledge about organ donation and transplantation, a lack of fear of organ trafficking, and attitudes towards donation.

## 1. Introduction

Although organ transplantation has established itself as a standard medical intervention, the number of individuals on waiting lists continues to surpass the availability of available organs. Compared to other medical specialties, transplantology is a field of particular social sensitivity, carrying ethical, medical, and socio-moral concerns . According to Caplan et al., transplant medicine is the only specialty in medical care that cannot exist without the participation and social acceptance of the public. It is an individual, whether alive or deceased, who makes it possible for their organs and tissues [1] to be used for transplantation [2]. In addition to the patient and the doctor performing the procedure, an organ is required from another person (living or deceased). The Transplantation Act and international regulations regulate organ transplantation from deceased and living donors in Poland. The latest statistics reveal a disproportionate gap between the needs and possibilities of identifying the donor and transplanting the organ [3].

In 2023, 756 potential deceased organ donors were reported to the Poltransplant Organizational and Coordination Center. In 556 cases (74%), organs were collected, which gives 15 donors per 1 million inhabitants. A total of 1898 organs were transplanted. Of these, 997 cadaveric kidneys were transplanted. At the end of 2023, 1193 active patients were waiting for kidney transplantation [3,4]. Organ transplantation from living donors in Poland accounts for 3–4% of the total number of kidney transplants [5]. For comparison, in the United States and Scandinavia, this figure is 40% [6]. The number of kidney transplants from living donors in Poland in 2023 was a record-breaking 78 [4]. As noted by Hermanowicz et al., the low prevalence of living kidney transplantation in Poland may be attributed to the absence of a national framework, coupled with high-quality systems in the domain of living kidney donation [7], as well as medical contraindications, withdrawal of previous agreement, and psychosocial reasons [8]. Unspecified kidney donation (UKD), also known as non-directed, anonymous, Good Samaritan, or altruistic donation, refers to the intention of an individual to donate a kidney to a recipient they do not know [9]. UKD can provide important support in the process of organ procurement for organ-sharing programs designed to bypass donor–recipient incompatibility. In Poland, there is no legal regulation for organ transplants from the UKD [10]. Altruistic kidney donation is exercised in various countries around the world, including Israel, the United States, the United Kingdom, Sweden, Canada, the Netherlands, Australia, and Spain [11]. Understanding factors that influence the willingness to donate kidneys to strangers is critical in promoting and expanding the living donor pool [12]. When considering the decision to become a living kidney donor, individuals must evaluate multiple factors, including the identity of the recipient and their own perceived safety concerns. Prospective donors should carefully assess the potential risks and benefits associated with kidney donation. Integral to this decision-making process are considerations such as the medical risks involved, an understanding of the implications of renal removal, readiness for the recovery period, and the impact on one’s ability to promptly resume professional responsibilities [13]. Also, some evidence gives information about long-term risk of hypertension and developing end-stage renal diseases in 1% of living donors 15 years after donation [14,15]. Although there are tangible advantages to the kidney transplant program, along with public support and clinical outcomes comparable to those of designated kidney donors, evidence suggests that transplant professionals approach unnamed kidney donors (UKDs) with a certain level of caution and skepticism [14]. Research has consistently shown that motivations for kidney donation are multifaceted, reflecting a blend of altruistic desires, personal experiences, and socio-cultural influences [10,16]. The initial investigation into healthcare professionals’ attitudes regarding unspecified kidney donors in the United Kingdom revealed significant findings that carry substantial clinical implications for the UKD program [17]. These findings underscore the necessity for a standardized approach to younger candidates that all transplant centers should consistently implement. Additionally, the research emphasizes the importance of applying thorough assessment criteria equally to both specified and unspecified donors, as well as advocating for a revised strategy in addressing donor expectations [12,18].

Healthcare workers have difficulty helping donors reach voluntary decisions, encounter resistance, and face barriers to confirming donors’ wishes [19]. Empathy towards others has been found to be an important factor in helping behavior, and the level of empathy is likely to be closely related to an individual’s willingness to donate an organ [20].

Self-efficacy has been extensively studied in the initiation and ongoing commitment to various health behaviors, including weight management, smoking cessation, and physical activity. Recent research within the European Donor Hospital Education Program has revealed that heightened self-efficacy among intensive care personnel is significantly associated with a reduction in the perceived challenges of soliciting organ donations. Despite growing interest in this area, there is still limited understanding of the influence of self-efficacy on individuals’ awareness, attitudes, and behaviors regarding organ donation [21,22,23,24]. Rochelle et al. find that subjective norms and self-efficacy strongly predict behavioral intention to donate, and subjective norms significantly mediate the relationship between self-efficacy and behavioral intention to donate [25].

We conducted a study to assess the willingness of Polish healthcare professionals employed in dialysis centers to be living kidney donors for a stranger. We also assessed the association of psychological variables with the willingness to donate a kidney to a stranger.

## 2. Materials and Methods

### 2.1. Study Design

We conducted a cross-sectional survey, from February 2023 to June 2024, of dialysis professionals from across Poland. Ethics approval for the study was granted according to the Declaration of Helsinki by the Bioethics Committee of the Andrzej Frycz Modrzewski Krakow University (decision no. KBKA /3/O/2023) and registered at ClinicalTrials.gov (ID: NCT05797337). Also, the guidelines of the Helsinki Declaration (World Medical Association, 2013), STROBE (Strengthening the Reporting of Observational Studies in Epidemiology) [26], as well as the General Data Protection Regulation [27], were followed.

### 2.2. Participants

The dialysis professionals included physicians and nurses from dialysis centers located in 16 voivodeships (voivodeship is Poland’s highest administrative division level). The study involved employees who had a communicative knowledge of Polish. Each participant received thorough information about the course and objectives of the study before it began. All participants provided informed consent. Out of the 1451 questionnaires delivered, 1093 (68%) were returned.

### 2.3. Measures

The study employed a diagnostic survey utilizing a questionnaire methodology. Questionnaires were distributed in both paper and online formats. Furthermore, employees at dialysis centers were encouraged via emails, advertisements in the trade press, or a dedicated QR code to participate, using the snowball sampling technique. A self-assessment questionnaire was utilized, which included a sociodemographic data sheet as well as inquiries about the respondents’ attitudes toward kidney transplantation, their knowledge in this field, and the educational methods implemented in the facilities where they provide care for dialysis patients. A structured questionnaire comprising various items was employed for this purpose. The questionnaire was adapted from a study on factors influencing informational support provided by dialysis staff to dialysis patients in their centers (including attitudes and knowledge about transplantation and willingness to undergo kidney transplantation) [28].

The study group, based on the willingness to be a living kidney donor to strangers, was divided into two groups: willing and unwilling. This was assessed by asking the respondents the following question: “The law on organ donation and transplantation in some countries around the world allows for the donation of a kidney to a stranger. What do you think about this?”

The first group included respondents who answered as follows:

(1) “I consider it a heroic act, worthy of admiration, I would also be willing to do it” (*n* = 90; 8%);
(2) “I think it is an attitude worth emulating, I could consider it after a thorough explanation of the consequences for my life and health” (*n* = 325; 30%).

The second group consisted of respondents who answered as follows:

(1) “I think it is a wonderful gesture, but I would never do it myself” (*n* = 407; 37%);
(2) “I think that such an act means making yourself “disabled”—you cannot live normally with one kidney” (*n* = 6; 0.5%);
(3) “I have no opinion’’ (*n* = 228; 21%);
(4) “Other” (*n* = 37; 3%).

Empathy was assessed using the Polish adaptation of the Interpersonal Reactivity Index (IRI) [29]. This instrument comprises 28 items divided into four distinct subscales (each consisting of seven items): Fantasy (IRI-Fs), which reflects the inclination to become emotionally involved with fictional characters; perspective taking (IRI-PT), indicating the capacity to understand others’ viewpoints; empathic concern (IRI-EC), representing the tendency to feel compassion and concern for people in distress; and personal distress (IRI-PD), which measures self-oriented feelings of discomfort in response to others’ suffering. Responses are rated on a 5-point Likert scale ranging from 0 (“does not describe me well”) to 4 (“describes me very well”). Higher scores on each subscale correspond to greater tendencies in the respective aspect of empathy [29].

The next standardized tool was the Polish version [26] of the Satisfaction with Life Scale [30]. This is a short 5-item instrument designed to measure global cognitive judgments of satisfaction with one’s life. Responses are given using a 7-point Likert scale, where 7 corresponds to I strongly agree, and 1 to I strongly disagree. The results were measured and presented as a general indicator of the feeling of satisfaction with life, ranging from 5 to 35 points (where 20 is considered neutral). The higher the score, the greater the sense of life satisfaction [30].

Self-esteem was assessed using the Rosenberg Self-Esteem Scale (RSES) [31], a widely used instrument for evaluating overall self-worth through an assessment of both positive and negative self-perceptions. The scale consists of 10 statements rated on a 4-point Likert scale, where 0 corresponds to “strongly agree” and 3 to “strongly disagree”. Half of the items (1, 2, 4, 6, and 7) are phrased positively, while the remaining items (3, 5, 8, 9, and 10) are worded negatively. The total score ranges from 0 to 30, with higher scores indicating greater self-esteem. Raw scores were subsequently converted into standardized units using the sten scale. The Polish version, adapted by Łaguna et al. [32], has demonstrated solid psychometric properties, with Cronbach’s alpha values ranging from 0.81 to 0.83.

To assess self-efficacy, the Generalized Self-Efficacy Scale (GSES), developed by Schwarzer and Jerusalem, was employed, using the Polish adaptation by Juczyński [33]. This scale comprises 10 items that collectively represent a single construct. Responses are scored according to a standard key, with interpretation based on sten norms. The Polish adaptation has shown good internal consistency, with a Cronbach’s alpha of 0.85.

### 2.4. Outcomes

The primary outcomes described the percentage of healthcare professionals willing to be a living kidney donor for a stranger. The secondary outcomes included factors associated with the willingness to be a living kidney donor for a stranger.

### 2.5. Data Analysis

The data was collected in Excel format and later imported into STATISTICA v.13 (TIBCO Software Inc., Kraków, Poland, 2017) for data quality checks and analysis. Categorical variables were described as absolute numbers and percentages, while the continuous variables were presented as medians and upper and lower quartiles. The test of normality of continuous variables was performed using the Shapiro–Wilk test. The chi-squared test was used for the association between categorical independent variables and the outcome variable. Differences between variables across groups for continuous variables were assessed using the Whitney test. Multiple logistic regression models were used to assess the effect of the independent variables (e.g., profession, perceived self-efficacy) on the willingness to be a living organ donor for a stranger. The construction of the multivariate model was based on independent variables, whose significance level *p* in the simple logistic analysis was no greater than 0.1. The best possible logistic regression model was obtained by a method called stepwise backward elimination. The goodness of fit of the final model was verified by the Hosmer–Lemeshow test (the model is a good fit to the data as *p* > 0.05). Nagelkerke’s R^2^ describes the proportion of variance in the outcome that the model successfully explains. The Wald test statistic was used to assess the contribution of individual predictors or the significance of individual coefficients for the reduced model. The 95% confidence interval (CI) was used to estimate the precision of the odds ratio (OR). All the statistical tests were performed at the 5% significance level. The results obtained from the various analyses are presented in tables.

## 3. Results

### 3.1. Sociodemographic Characteristics

**Figure 1:**
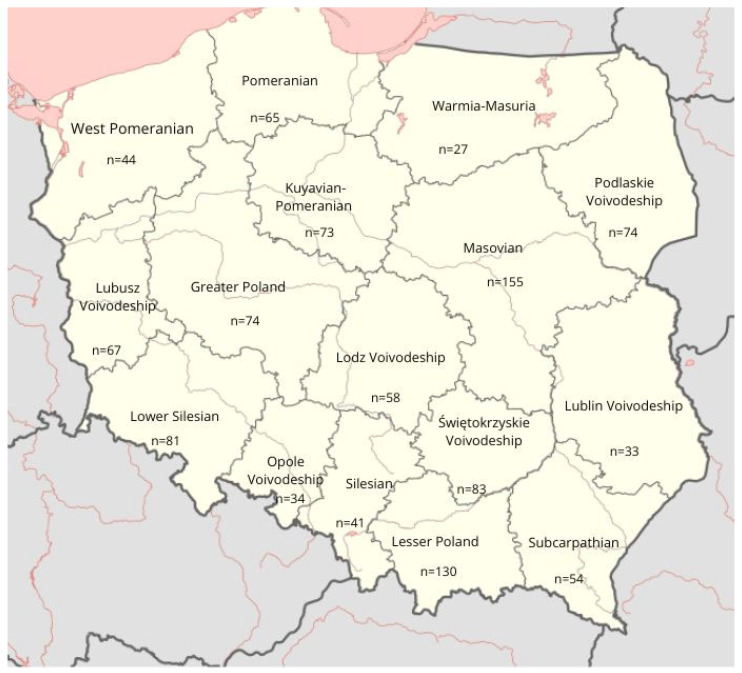
Map demonstrating the distribution of participation across Poland.

Out of the 1451 questionnaires delivered, 1093 (68%) were returned. Ultimately, data from 1093 healthcare professionals was included in the analysis because 57 questionnaires were incomplete and one was from a person living with one kidney. The largest number of respondents came from the Masovian voivodeship (*n* = 155; 14%) and the Lesser Poland voivodeship (*n* = 130; 12%), and the fewest from the Warmian-Masurian voivodeship (*n* = 27; 2%) (Figure 1).

Table 1 presents the association between sociodemographic characteristics and the willingness to donate a kidney to a stranger. In total, 415 individuals (38% of all respondents) supported living organ donation. Individuals in this group differed significantly from those who did not consider this option (*n* = 678; 62%) only in terms of their profession—nurses were more willing to donate than physicians. Notably, nurses were significantly older than physicians (median age: 50 [45; 55] vs. 48 [37; 55]; Z = 2.63; *p* = 0.008) and had been in their jobs longer (20 [8; 28] vs. 15 [5; 23] years; Z = 4.06; *p* < 0.0001).

### 3.2. Psychological Variables

Table 2 presents the descriptive statistics of all the study’s psychological variables. The medians of empathy, life satisfaction, and self-esteem are similar in the willing and unwilling groups (*p* > 0.05). Statistically significant differences were found only between the two groups in terms of perceived self-efficacy (30 [28; 32] vs. 29 [27; 32]; *p* = 0.039). A weak positive correlation was found between perceived self-efficacy and life satisfaction (R = 0.34; *t* = 12.01; *p* < 0.0001), self-esteem (R = 0.38; *t* = 13.41; *p* < 0.001), and perspective taking of empathy (R = 0.15; *t* = 5.02; *p* < 0.0001). On the other hand, personal distress associated with empathy was negatively correlated with perceived self-efficacy (R = −0.25; *t* = −8.44; *p* < 0.0001).

Compared to physicians, nurses had significantly lower median scores for life satisfaction (23 [20; 26] vs. 25 [22; 29]; Z = −6.29; *p* < 0.0001), self-esteem (20 [18; 22] vs. 22 [18; 25]; Z = −4.29; *p* < 0.0001), and perceived self-efficacy (30 [27; 32] vs. 30 [28; 33]; Z = −2.15; *p* = 0.032). Moreover, there is a statistically significant difference in the median level of the personal distress subscale (14 [12; 17] vs. 12 [8; 14]; Z = 9.29; *p* < 0.0001) and the fantasy subscale (14 [12; 17] vs. 14 [10; 17]; Z = 1.99; *p* = 0.046) between nurses and physicians. This means that nurses are more likely to feel pain and discomfort in response to people suffering from end-stage renal disease and to imagine what difficulties such a patient may be experiencing.

### 3.3. Attitude Toward Donating Cells, Tissues, and Organs

Health professionals who considered the possibility of kidney donation to a stranger in the future more often registered with the bone marrow donor bank (22% vs. 17%; *p* = 0.026), declared support for legalization of unspecified living kidney donation in Poland (19% vs. 10%; *p* < 0.0001), and were less likely to believe in the risk of organ trafficking (6% vs. 16%; *p* < 0.0001) than those who were against the idea (Table 3). It is worth noting that over 60% of all respondents were willing to donate one of their organs after their death. More than half of the respondents (52–54%) would agree to donate an organ from a deceased close family member.

### 3.4. Willingness to Be a Living Kidney Donor or Accept Kidney Transplantation

Respondents from the willing group were more likely to envisage donating a kidney to a child (90% vs. 83%), husband or wife (62% vs. 45%), siblings (58% vs. 47%), parent (57% vs. 40%), friend (24% vs. 8%), another member of the family (22% vs. 9%), and cohabitee (20% vs. 13%) in comparison with the unwilling group. They also more often supported their close family donating a kidney to a family member (70% vs. 64%), a cohabiting partner or friend (46% vs. 34%), or to a stranger (27% vs. 11%) (Table 4).

The survey results indicate that 8–50% of respondents would agree to accept a kidney from a living donor as part of the treatment for end-stage renal disease. Those who were willing to donate a living organ to strangers would be more likely to accept a kidney from, for example, a husband or wife (50% vs. 30%), siblings (38% vs. 29%), or a stranger (34% vs. 24%), than respondents from the unwilling group. However, for half of the respondents, accepting a kidney from a deceased donor would also be a good option (Table 4).

### 3.5. Factors Associated with the Willingness to Donate a Kidney to a Stranger

The factors associated with the willingness of healthcare professionals to donate a kidney to a stranger are presented in Table 5. Nurses (vs. physicians) and those who supported the regulation of unspecified living kidney donation in Poland, did not fear the risk of organ trafficking, and would donate a kidney to a husband/wife or friend and accepted kidney transplantation from a husband/wife were more likely to donate a kidney to a stranger. Furthermore, respondents who accepted a loved one’s decision to donate a kidney to a stranger were significantly more willing to donate a kidney to such a person themselves. Perceived self-efficacy correlated positively with the willingness to donate a kidney to a stranger.

Nagelkerke’s R^2^ suggests that the model explains 20% of the variation in the outcome.

## 4. Discussion

In the last twenty years, various actions have been taken to broaden the living donor pool, one of which includes the introduction of unspecified kidney donation [34,35,36]. We first conducted a national cross-sectional study in Poland to assess the willingness of Polish healthcare professionals employed in a dialysis center to be a living kidney donor for a stranger.

### 4.1. The Willingness to Be a Living Kidney Donor to a Stranger Among Polish Healthcare Professionals

Our study revealed that 38% (*n* = 415) of individuals supported unspecified living organ donation. Individuals in this group differed significantly from those that did not consider this option (*n* = 678; 62%) only in terms of the profession performed—nurses were more willing to donate than physicians. It is worth noting that nurses were significantly older and had been in their job longer than physicians. Maple et al. found that most respondents reported a positive experience with UKDs (81.7%) while [36] not finding significant differences between professions’ attitudes [37]. Findings from the United Kingdom indicate that kidney transplant professionals view UKD differently than designated donation. The potential harm inflicted on patients on the waiting list, who are denied transplantation opportunities due to restrictions on UKD, does not seem to mitigate the concerns associated with UKD itself [12]. Zuchowski et al. investigated the attitudes of UK healthcare staff toward UKD [18]. The qualitative study found that many participants suggested that a minimum age limit should be established for potential donors. Concerns were also raised about the psychological stability of UKDs, with their motivations being complex or ambiguous. There was also a clear need to manage expectations of UKDs, particularly in terms of communication with recipients. Finally, the results suggested that some healthcare professionals felt that their personal views had no influence on the voluntary withdrawal of UKD candidates [18]. When examining physicians’ attitudes towards living kidney donation, Trachtman et al. discovered in their study that nephrologists overwhelmingly endorse living kidney donation as a feasible medical option, with nearly all expressing support for healthy, medically cleared patients who wish to participate. Although overall support remained high, nephrologists were notably less enthusiastic about accepting friends and relatives to donate a kidney during their lifetime. The level of acceptance dropped even more when it came to donating to non-relatives [37].

The study’s findings suggest that transplant teams should encourage donors to discuss their donation intentions with their families early on to address any potential concerns. In addition, potential donors should be connected with previous donors for emotional support. Participants noted negative attitudes among some healthcare providers toward unspecified donors, likely due to concerns about exposing healthy individuals to risks that distinguish them from other surgical patients [38,39].

### 4.2. Factors Associated with the Willingness to Be a Living Kidney Donor to a Stranger

Our results revealed that nurses (vs. physicians) and those who supported the legalization of unspecified living kidney donation in Poland, and did not fear the risk of organ trafficking, and would donate a kidney to a husband/wife or friend, and accepted kidney transplantation from a husband/wife were more likely to donate a kidney to a stranger. Furthermore, respondents who accepted a loved one’s decision to donate a kidney to a stranger were significantly more willing to donate a kidney to such a person themselves. Perceived self-efficacy was positively associated with the willingness to donate a kidney to a stranger.

Clarke et al. emphasize the importance of social relationships in the process and outcomes of UKD, noting that donors experience both benefits and stresses. While the donation process offers personal benefits, it also leads to conflicting views about the acceptability of their donation, affecting both their personal lives and interactions with healthcare services. The results are contextualized within the broader literature on UKD and altruism, highlighting their implications for clinical practice [40].

Zuchowski et al., in their qualitative study, found that the participants questioned the role of altruism as a motivator for UKD. They remark that motivations are multifaceted or ambiguous, as it can sometimes be challenging to determine whether candidates are entirely selfless or have self-interested motives [18]. Those who believe that bone marrow donations are safe would support legalizing such donations, as would respondents who support family members donating kidneys to strangers or becoming deceased organ donors. However, UKD does not have support from those who fear the risks of organ trafficking. Therefore, any legal changes to allow UKD must impose severe penalties to prevent organ sales, human exploitation, and human trafficking [41].

In our study, we found that perceived self-efficacy was positively associated with willingness to donate a kidney to a stranger. In their study, Janicka et al. found that most respondents had low self-efficacy and negative attitudes toward donating organs from healthy, living donors to save the lives of those in need. In addition, they would not consent to donate an organ from a deceased loved one [42].

Empathy is one of the factors that can influence the decision to make an organ donation [43,44,45]. Milaniak et al. found that the higher scores in the perspective-taking subscale were found to be significantly associated with fear of surgical treatment as the reason for refusing to give one’s consent to a living donation [45]. In our study, we did not find a difference between willing and unwilling medical staff. However, nurses are more likely to feel pain and discomfort in response to people suffering from end-stage renal disease and to imagine the difficulties such a patient may be experiencing.

### 4.3. Strengths and Limitations

The study’s strengths lie in its large sample size and the use of standardized instruments, marking it as the first nationwide investigation on this topic. However, a limitation is that the self-assessment questionnaire employed was not validated, and the sample structure could not be calculated due to insufficient data on the number of nephrologists and nurses working in dialysis centers across the country.

## 5. Conclusions

Less than half of healthcare professionals supported unspecific living organ donation in Poland, and nurses were more willing to donate than physicians. The factors supporting the decision generally included knowledge about organ donation and transplantation, not fearing the risk of organ trafficking, and attitudes towards donation. Perceived self-efficacy correlated positively with the willingness to donate a kidney to a stranger. Due to the lack of a legal framework, infrastructure, and resources to support unspecific living kidney donation, it is essential to discuss the implementation of policies from other European countries with key players. It is essential to strengthen training and ensure consistency among all members of the multidisciplinary teams at dialysis centers who prepare candidates for kidney transplants and discuss the option of receiving a kidney from either a living or deceased donor. Such measures can enhance the donation experience for all parties involved and lead to greater acceptance of unspecified donations as a vital component of the kidney transplant program.

## Figures and Tables

**Table 1:** Association of sociodemographic data with the willingness to donate a kidney to a stranger.

	Willingness to Donate a Kidney to a Stranger			
	Willing *n* = 415	Unwilling *n* = 678	Statistics Value	*p*-Value
Age (years)	50 [41; 55]	50 [43; 55]	Z = 0.61	0.539
Time in Job (years)	19 [7; 27]	20 [8; 27]	Z = −0.16	0.871
Gender				
- Female	376 (91)	587 (87)	χ^2^ = 3.98	0.046
- Male	39 (9)	91 (13)		
Place of residence				
- City	300 (72)	515 (76)	χ^2^ = 1.83	0.176
- Village	115 (28)	163 (24)		
Married or in a committed relationship	310 (75)	511 (75)	χ^2^ = 0.06	0.804
Having children	321 (77)	536 (79)	χ^2^ = 0.44	0.505
Having siblings	370 (89)	583 (86)	χ^2^ = 2.31	0.128
The dialysis center is the main workplace	296 (71)	484 (71)	χ^2^ = 0.0005	0.983
Profession				
- Nurse	353 (85)	497 (73)	χ^2^ = 20.58	<0.0001 *
- Physician	62 (15)	181 (27)		
Specialization program completed	184 (44)	312 (46)	χ^2^ = 0.29	0.588
Educational degree of nurses (*n* = 850)				
- Master of Science in Nursing	119 (34)	156 (31)	χ^2^ = 0.59	0.745
- Bachelor in Nursing	114 (32)	162 (33)		
- Registered Nurse	120 (34)	179 (36)		

Age and time in job are presented as median [upper and lower quartile]. Categorical variables are presented as absolute numbers and percentages. * statistically significant.

**Table 2:** Association of psychological variables with the willingness to donate a kidney to a stranger.

Variable	Willingness to Donate a Kidney to a Stranger				
	Willing *n* = 415	Unwilling *n* = 678	Statistics Value	*p*-Value	Cronbach’s
Life satisfaction	24 [20; 27]	23 [20; 27]	*Z* = 1.003	0.316	0.86
Self-esteem	20 [18; 23]	20 [18; 23]	*Z* = −0.16	0.873	0.83
Perceived self-efficacy	30 [28; 32]	29 [27; 32]	*Z* = 2.06	0.039 *	0.86
Empathy subscales					
- Perspective taking	17 [15; 19]	17 [14; 19]	*Z* = 1.32	0.186	
- Empathic concern	17 [15; 20]	17 [14; 21]	*Z* = 1.64	0.099	
- Personal distress	14 [12; 16]	14 [11; 16]	*Z* = 0.78	0.434	
- Fantasy	14 [12; 18]	14 [11; 17]	*Z* = 1.25	0.210	

Psychological variables are presented as median [interquartile range]. * statistically significant.

**Table 3:** Association of attitude towards transplantation of cells, tissues, and organs with the willingness to donate a kidney to a stranger.

Variable	Willingness to Donate a Kidney to a Stranger			
	Willing *n* = 415	Unwilling *n* = 678	Statistics Value	*p*-Value
Registered with the bone marrow donor bank	93 (22)	115 (17)	χ^2^ = 4.95	0.026 *
A bonemarrow transplant is safe	186 (45)	314 (46)	χ^2^ = 0.23	0.630
Blood donor at least once in life	100 (24)	167 (25)	χ^2^ = 0.04	0.842
Blood donation is safe	250 (60)	384 (57)	χ^2^ = 1.31	0.252
Donating one’s organs after death	274 (66)	434 (64)	χ^2^ = 0.46	0.499
Consent to donating an organ from a deceased close family member	223 (54)	351 (52)	χ^2^ = 0.39	0.528
Asking for the family’s consent to donate an organ from a deceased close family member	147 (35)	224 (33)	χ^2^ = 0.65	0.419
Support for legalization of unspecified living kidney donation in Poland	81 (19)	65 (10)	χ^2^ = 21.94	<0.0001 *
Organ trafficking risk	26 (6)	109 (16)	χ^2^ = 22.89	<0.0001 *

Categorical variables are presented as absolute numbers and percentages. * statistically significant.

**Table 4:** The willingness to donate a kidney to another human and to accept kidney transplantation.

Variable	Willingness to Donate a Kidney to a Stranger			
	Willing *n* = 415	Unwilling *n* = 678	Statistics Value	*p*-Value
Willingness to donate a kidney				
- Parent	237 (57)	273 (40)	χ^2^ = 29.34	<0.0001 *
- Child	372 (90)	566 (83)	χ^2^ = 8.02	0.005 *
- Siblings	243 (58)	317 (47)	χ^2^ = 14.3	0.0001 *
- Husband or wife	257 (62)	303 (45)	χ^2^ = 30.61	<0.0001 *
- Cohabitee	85 (20)	91 (13)	χ^2^ = 9.49	0.002 *
- Another member of the family	91 (22)	60 (9)	χ^2^ = 36.98	<0.0001 *
- Friend	101 (24)	54 (8)	χ^2^ = 56.70	<0.0001 *
Willingness to accept kidney transplantation				
- Parent	146 (35)	200 (29)	χ^2^ = 3.84	0.05
- Child	65 (16)	66 (10)	χ^2^ = 8.58	0.003 *
- Siblings	157 (38)	198 (29)	χ^2^ = 8.74	0.003 *
- Husband or wife	209 (50)	236 (35)	χ^2^ = 25.79	<0.0001 *
- Cohabitee	95 (23)	96 (14)	χ^2^ = 13.61	0.0002 *
- Another member of the family	81 (19)	67 (10)	χ^2^ = 20.42	<0.0001 *
- Friend	81 (19)	58 (8)	χ^2^ = 27.88	<0.0001 *
- Stranger	143 (34)	160 (24)	χ^2^ = 15.15	<0.0001 *
- Dead donor	197 (47)	353 (52)	χ^2^ = 2.17	0.140
Complete acceptance of a loved one’s decision to donate a kidney to a stranger				
- Family member	292 (70)	433 (64)	χ^2^ = 4.86	0.027 *
- Cohabitee or friend	191 (46)	228 (34)	χ^2^ = 16.73	0.00004 *
- Stranger	113 (27)	74 (11)	χ^2^ = 48.31	<0.0001 *

Categorical variables are presented as absolute numbers and percentages. * statistically significant.

**Table 5:** Factors associated with the willingness to donate a kidney to a stranger.

Variable	*B*	SE (B)	Wald Test	*p*	OR (CI 95%)
**Simple logistic regression**					
Nurse^Reference: Physician^	0.36	0.08	20.07	0.000	1.44 (1.23–1.69)
Perceived self-efficacy	0.03	0.01	3.08	0.079	1.03 (1.00–1.06)
Registered with the bone marrow donor bank	0.17	0.08	4.93	0.026	1.19 (1.02–1.38)
Support for legalization of unspecified living kidney donation in Poland	0.41	0.09	21.15	0.000	1.51 (1.17–1.80)
Organ trafficking risk	−0.53	0.11	21.34	0.000	0.59 (0.47–0.74)
Willingness to donate a kidney					
- Parent	0.34	0.06	29.01	0.000	1.40 (1.24–1.59)
- Child	0.27	0.10	7.89	0.005	1.31 (1.08–1.58)
- Siblings	0.24	0.06	14.26	0.0002	1.27 (1.12–1.43)
- Husband or wife	0.35	0.06	30.24	0.000	1.42 (1.25–1.61)
- Cohabitee	0.25	0.08	9.38	0.002	1.29 (1.10–1.52)
- Another member of family	0.53	0.09	34.87	0.000	1.70 (1.43–2.03)
- Friend	0.67	0.09	51.91	0.000	1.93 (1.61–2.30)
Willingness to accept kidney transplantation					
- Child	0.27	0.09	8.43	0.004	1.31 (1.09–1.58)
- Siblings	0.19	0.07	8.70	0.003	1.21 (1.07–1.38)
- Husband or wife	0.32	0.06	25.53	0.000	1.38 (1.22–1.56)
- Cohabitee	0.29	0.08	13.39	0.0003	1.34 (1.15–1.57)
- Another member of the family	0.40	0.09	19.75	0.000	1.49 (1.25–1.77)
- Friend	0.48	0.09	26.54	0.000	1.61 (1.34–1.93)
- Stranger	0.27	0.07	15.01	0.0001	1.30 (1.14–1.49)
Complete acceptance of a loved one’s decision to donate a kidney to a stranger					
- Family member	0.15	0.07	4.85	0.028	1.16 (1.02–1.32)
- Cohabitee or friend	0.26	0.06	16.61	0.000	1.30 (1.14–1.47)
- Stranger	0.56	0.08	45.61	0.000	1.75 (1.49–2.05)
**Multiple logistic regression model**					
Nurse^Reference: Physician^	0.53	0.09	33.18	0.000	1.69 (1.42–2.03)
Perceived self-efficacy	0.03	0.02	4.44	0.035	1.04 (1.00–1.07)
Support for legalization of unspecified living kidney donation in Poland	0.27	0.10	7.41	0.006	1.32 (1.08–1.60)
Organ trafficking risk	−0.49	0.12	16.71	0.000	0.61 (0.48–0.77)
Willingness to donate a kidney					
- Husband or wife	0.21	0.08	7.12	0.008	1.24 (1.06–1.45)
- Friend	0.54	0.10	27.85	0.000	1.72 (1.41–2.11)
Willingness to accept kidney transplantation from husband or wife	0.18	0.08	4.84	0.028	1.19 (1.02–1.39)
Complete acceptance of the decision to donate a kidney to a stranger by someone close to you	0.44	0.09	23.49	0.000	1.55 (1.30–1.85)

B: regression coefficient; SE: standard error; OR: odds ratio; and CI: confidence interval. Multivariable logistic regression model: R^2^ Nagelkerke  =  0.20; Hosmer–Lemeshow = 7.47; *p* = 0.487.

## Data Availability

The authors will make the raw data on which the conclusions in this article are based available without undue reservation upon request.
